# Observation of aldicarb hydrolysis by a cocaine hydrolase

**DOI:** 10.1042/BSR20254091

**Published:** 2026-04-22

**Authors:** Johnathan E. LeSaint, Daniel J. Peter, Huimei Wei, Shawn Park, Chang-Guo Zhan, Fang Zheng

**Affiliations:** 1 Molecular Modeling and Biopharmaceutical Center, College of Pharmacy, University of Kentucky, 789 South Limestone Street, Lexington, KY 40536, U.S.A.; 2 Department of Pharmaceutical Sciences, College of Pharmacy, University of Kentucky, 789 South Limestone Street, Lexington, KY 40536, U.S.A.

**Keywords:** Carbamate, cholinergic warfare, cocaine hydrolase, esterase, hydrolysis, pesticide

## Abstract

Aldicarb is a carbamate pesticide used for pest control in agriculture. As a fast-acting acetylcholinesterase inhibitor, aldicarb interferes with the nervous system by preventing the breakdown of acetylcholine. Aldicarb could be used as a chemical-warfare agent to cause mass casualty incidents. There is no specific FDA-approved medication for aldicarb detoxification. Our previous study revealed that an Fc-fused butyrylcholinesterase (BChE) mutant, known as CocH3-Fc(M3), can be inhibited rapidly by aldicarb and that the aldicarb-inhibited enzyme CocH3-Fc(M3) can be reactivated spontaneously, suggesting that CocH3-Fc(M3) may hydrolyze aldicarb. However, the suggested CocH3-Fc(M3)-catalyzed hydrolysis of aldicarb was not confirmed experimentally in the previous study. In the present study, by developing an LC-MS/MS method to detect and quantify aldicarb and aldicarb oxime concentrations, we were able to directly observe the CocH3-Fc(M3)-catalyzed aldicarb hydrolysis for the first time, confirming that CocH3-Fc(M3) indeed has the desirable catalytic activity for aldicarb hydrolysis and may be considered as the first aldicarb hydrolase identified so far. Further, we carried out Michaelis–Menten kinetic analysis on the CocH3-Fc(M3)-catalyzed aldicarb hydrolysis and determined the catalytic parameters (*k*_cat_ *=* 0.060 min^−1^, *K*_M_ *=* 2.5 μM, and *k*_cat_/*K*_M_ *=* 2.4 × 10^4^ min^−1^ M^−1^) at 37°C. The obtained kinetic parameters at 37°C will be valuable for further *in vivo* studies and translational research using CocH3-Fc(M3) and for designing more potent enzymes to hydrolyze aldicarb in the future. Additionally, the LC-MS/MS method developed in this study may serve as a valuable tool to accurately detect aldicarb and its reaction products in future food and environmental safety control efforts and aldicarb-related toxicology studies.

## Introduction

Aldicarb is a carbamate pesticide used for pest control in agriculture [1]. As a potent acetylcholinesterase (AChE) inhibitor, aldicarb interferes with the nervous system by preventing the breakdown of acetylcholine. The buildup of acetylcholine at synapses disrupts nerve function, resulting in a range of toxicity symptoms [1], such as tremor, muscle weakness, lethargy, salivation, lacrimation, fasciculation, mastication, convulsion, and death. As the most toxic carbamate pesticide, aldicarb could also be used as chemical-warfare agent to cause mass casualty incidents [1]. Aldicarb has been classified as a Chemical of Concern by the U.S. Department of Homeland Security, and it is important for the chemical and biological defense to develop effective medical countermeasures and therapeutic interventions for the aldicarb exposures [2]. However, there is no specific FDA-approved medication available for aldicarb detoxification. Currently clinical options of treatment for aldicarb poisoning use unapproved medical products that manage aldicarb poisoning symptoms, such as atropine interacting with cholinergic muscarinic receptors to reduce saliva and fluid in the respiratory tract, diphenhydramine to counteract the effects of aldicarb on nicotinic receptors, and diazepam to control seizures, *etc* [3–5]. Other supportive treatments, including fluids, may also be supplied. According to the FDA, unapproved products carry potential risks. It is highly desired to develop a new, effective treatment strategy for aldicarb poisoning [1].

Aldicarb can be detoxified and eliminated slowly from the body after the aldicarb exposure [6]. For example, in a case report of aldicarb poisoning [5], urine aldicarb levels were monitored at various time points after admission to an intensive care unit (ICU), and significant aldicarb levels were detected at 61.5 h after admission to the ICU. The condition of the patient gradually improved on days 2 and 3 while the aldicarb levels gradually decreased, and the patient was discharged at 80 h after admission to the ICU [5].

**Figure 1:**
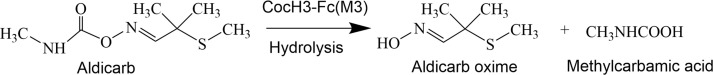
Reaction scheme CocH3-Fc(M3)-catalyzed hydrolysis of aldicarb to produce AO and methylcarbamic acid.

The natural detoxification process of aldicarb in humans is that aldicarb is metabolized into various metabolites, primarily aldicarb sulfoxide as well as (in lower quantities) aldicarb sulfone, which are then further decomposed (by hydrolysis or dehydration) and excreted [7]. Additionally, aldicarb and its metabolites have also been detected in environmental samples such as soil and water [8]. There have been intensive studies on aldicarb-related toxicology and food and environmental safety, particularly in the detection of aldicarb [9–21]. Notably, as one of the aldicarb metabolites, aldicarb sulfoxide produced *via* oxidation is still toxic [22]. Thus, accelerating the hydrolysis of aldicarb into non-toxic aldicarb oxime (AO) using an exogenous enzyme (as shown in Figure 1) [23] could be an effective strategy for aldicarb detoxification.

Butyrylcholinesterase (BChE) is a type of cholinesterase enzyme like AChE. BChE is produced in liver, mainly present in plasma, and involved in the breakdown of neurotransmitters in plasma [24]. It is well known, BChE plays an important role in detoxifying various xenobiotic substances. BChE and its mutants have a long-term history of investigation for therapeutic use, acting as a bioscavenger to detoxify organophosphate (OP) nerve agents [24] and as candidates for treatment of cocaine overdose and abuse due to their potent *in vivo* efficacy, safety, and long-term stability [25–34]. Most recently, we discovered that an Fc-fused BChE mutant, known as CocH3-Fc(M3) (i.e. the A199S/F227A/S287G/A328W/Y332G mutant of human BChE truncated after amino acid 529 and fused to the N-terminus of a triple A1V/D142E/L144M mutant of Fc portion of human IgG1), which was originally designed and discovered in our lab as a long-acting and highly efficient cocaine hydrolase, has a ∼4-fold improved binding affinity (*K*_i_ = 13.1 μM) with aldicarb compared to wild-type AChE and wild-type BChE (IC_50_ = ∼67 μM) [23]. It has been demonstrated that CocH3-Fc(M3) can be inhibited by aldicarb more rapidly with a 9-28-fold improved bimolecular rate constant (*k* = 2.21 × 10^4^ M^−1^ min^−1^) compared to that of the wild-type enzymes based on the *in vitro* activity data, and CocH3-Fc(M3) can also effectively rescue mice injected with a lethal dose of aldicarb (0.75 mg/kg, LD_100_) based on the *in vivo* activity data [23]. More interestingly, the *in vitro* activity data also revealed that the aldicarb-inhibited enzyme CocH3-Fc(M3) may be reactivated spontaneously [23], leading us to assume that CocH3-Fc(M3) may not only bind with and be inhibited by aldicarb, but also degrade aldicarb through hydrolysis as proposed in Figure 1. However, the assumed CocH3-Fc(M3)-catalyzed hydrolysis of aldicarb was not confirmed analytically in the previously reported study.

Here, we report the first direct observation of aldicarb hydrolysis catalyzed by CocH3-Fc(M3) by using an LC-MS/MS method to detect and quantify aldicarb and its hydrolysis product, AO (depicted in Figure 1), during the reaction process and the first Michaelis–Menten kinetic analysis for this promising enzyme against aldicarb.

## Results

### Calibration curves obtained for the LC-MS/MS method to quantify ALD and AO

**Figure 2:**
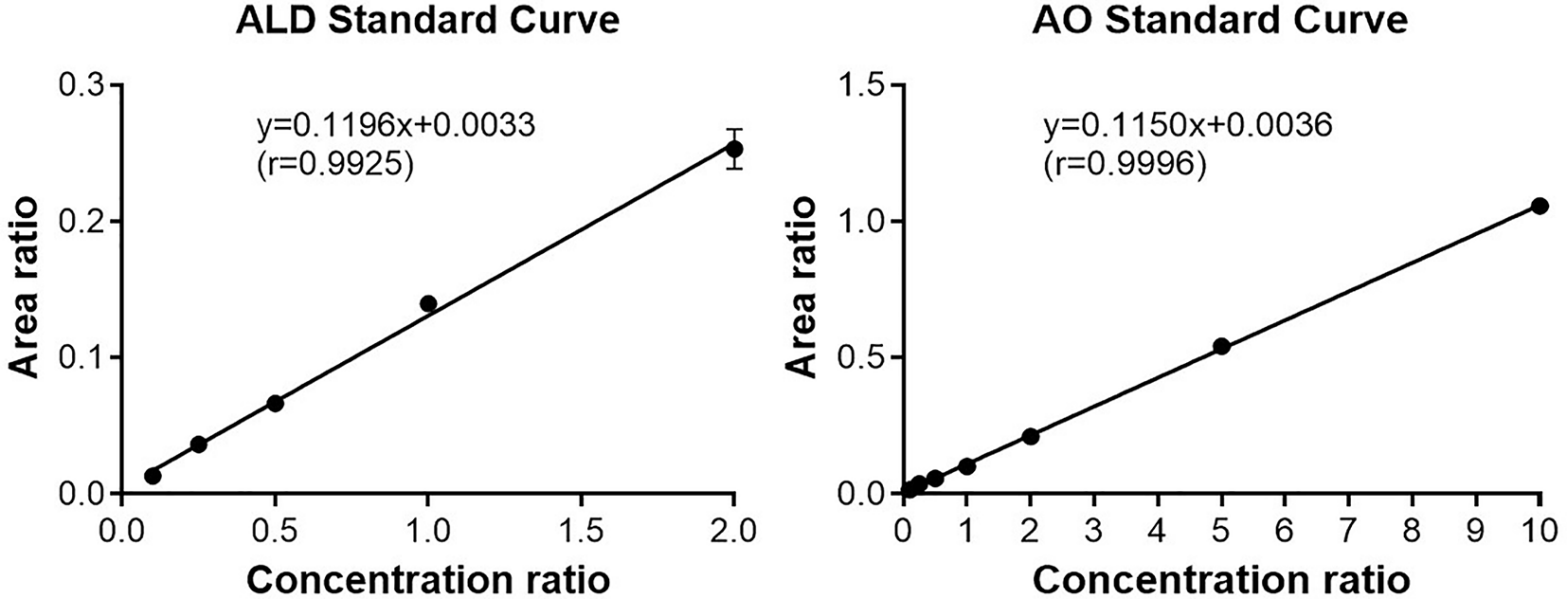
Calibration curves Calibration curves for calculating the concentrations of aldicarb (ALD) and AO using the developed LC-MS/MS protocol.

To quantify aldicarb (ALD) and AO, we first established an LC-MS/MS method with the optimized experimental conditions. The calibration standard curves for analytes ALD and AO detections using the isotopically labeled internal standard (IS) are summarized in Table 1. Each calibration curve was established by plotting the ratio of the peak area for analyte to that for IS (y-axis) as a function of the ratio of the analyte concentration to IS concentration (x-axis) (Figure 2). Due to the difficulty of sourcing isotopically labeled AO, we tested aldicarb-d3 (ALD-d3) or aldicarb sulfone-d3 (ASF-d3) as IS instead. For aldicarb, using ALD-d3 or ASF-d3 as its IS generated similarly satisfactory calibration curves (*r* >0.98 with either ALD-d3 or ASF-d3). However, while using ALD-d3 as the IS allowed for a reasonable linearity range (100–2000 ng/ml) for both aldicarb and AO, using ASF-D3 as the IS allowed for a higher upper limit of quantification (ULOQ) (10000 ng/ml) for the reaction product AO. ASF-d3 also functioned better as an IS due to its stronger signal intensity and good linear relationship between mass spectrometry signal and concentration in this method for both ALD and AO. So, at the end, we chose to use ASF-d3 as a common IS for quantifying both aldicarb and AO. The limit of detection (LOD) of the method was 10 ng/ml and 20 ng/ml for ALD and AO, respectively. The lower limit of quantification (LLOQ, i.e. the lowest concentration of analytes) with an accuracy within 20% for both ALD and AO was 100 ng/ml. For the generated calibration curves, the correlation coefficient (r) is >0.99 with an accuracy within ± (5–20)% for all concentration points within the range of the calibration curves.

**Table 1:** Linearity, regression diagnostics, LOD, and LLOQ

Compound	Linearity range (ng/ml)	Internal standard (ng/ml)	Slope	Intercept	*r*	LOD (ng/ml)	LLOQ (ng/ml)
ALD	100–2000	ASF-d3(1000)	0.120	0.0033	0.993	10	100
AO	100–10000	ASF-d3(1000)	0.115	0.0036	1.000	20	100

### Catalytic parameters of CocH3-Fc(M3) for hydrolysis of ALD

**Figure 3:**
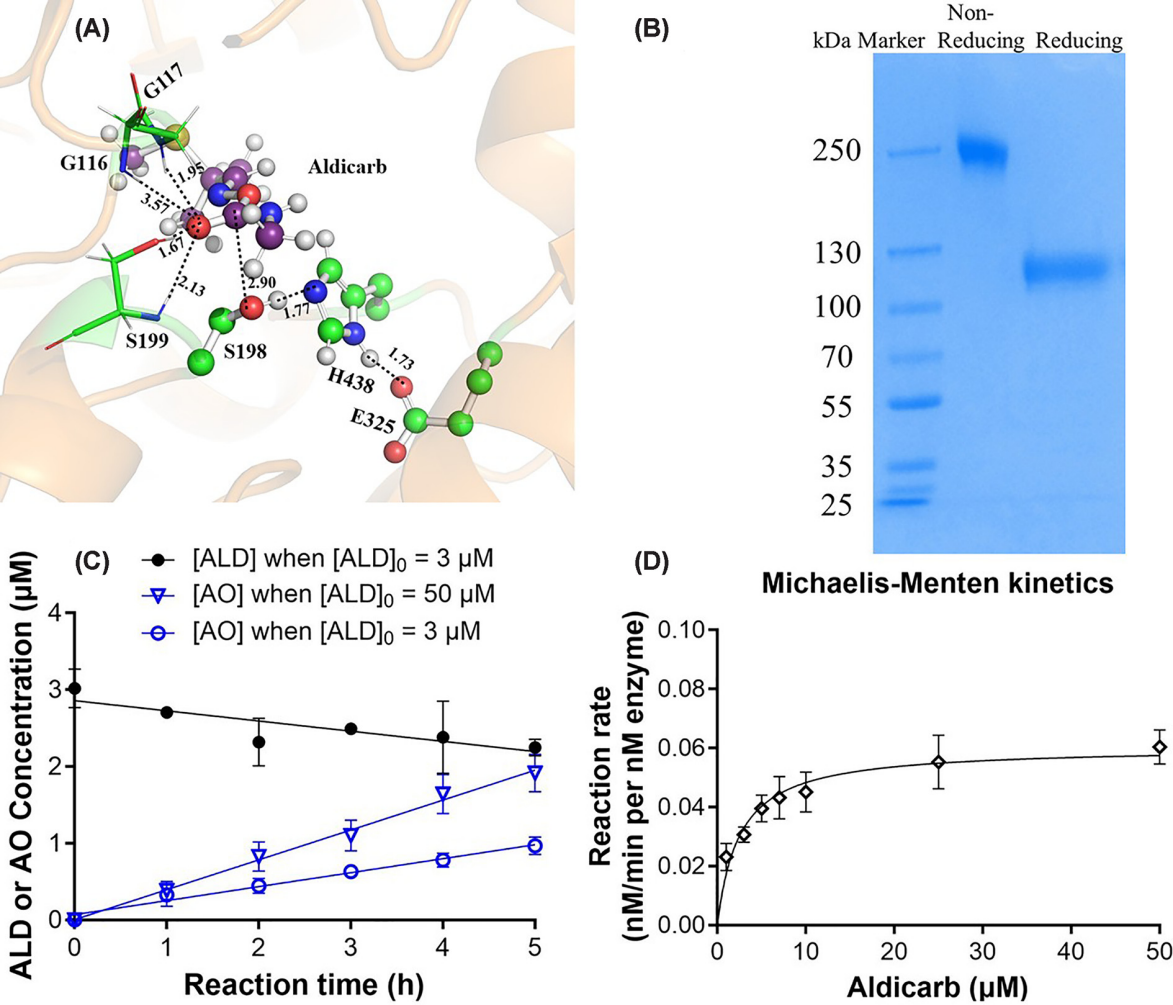
CocH3-Fc(M3)-catalyzed hydrolysis of ALD (**A**) Binding mode of CocH3 with aldicarb based on computational modeling (23), predicting that aldicarb binds to the active site of CocH3 ready for the catalytic hydrolysis. (**B**) Coomassie Blue-stained SDS electrophoresis gel of the purified CocH3-Fc(M3) protein (protein molecular weight marker, non-reducing, and reducing lanes). (**C**) Time-dependent aldicarb oxime (AO) concentrations [AO] in 0.1 μM CocH3-Fc(M3)-catalyzed hydrolysis of 3 or 50 μM aldicarb (ALD). Depicted in panel C is also time-dependent aldicarb (ALD) concentration when the initial aldicarb concentration (at time 0) [ALD]_0_ = 3 μM; the aldicarb concentrations [ALD] associated with [ALD]_0_ = 50 μM could not be quantified because they were out of the ALD linearity range (see Table 1) of the LC-MS/MS protocol. (**D**) Michaelis–Menten kinetic analysis on CocH3-Fc(M3) for aldicarb hydrolysis in which all *in vitro* activity measurements were performed in triplicate with a constant enzyme concentration of 0.1 μM and a reaction time of 5 h.

Using the established LC-MS/MS method, we measured the concentrations of aldicarb (ALD) and its hydrolysis product—AO—during reaction with 0.1 μM CocH3-Fc(M3) for determination of reaction rates for CocH3-Fc(M3)-catalyzed aldicarb hydrolysis under various initial aldicarb concentrations. The obtained kinetic data are shown in Figure 3. Depicted in Figure 3C are the time-dependent concentrations of AO—the product of aldicarb hydrolysis—at two representative initial aldicarb concentrations (3 and 50 μM). The data in Figure 3C revealed that (a) enzyme CocH3-Fc(M3) indeed hydrolyzed aldicarb, which is consistent with the computational insight (Figure 3A), and (b) the hydrolysis product concentration [AO] linearly increased over 5 h at both initial aldicarb concentrations, which was further confirmed by the decrease of aldicarb concentration for [ALD]_0_ = 3 μM, although detecting concentration variation for [ALD]_0_ = 50 μM was out of the ALD calibration curve range. So, the reaction rate (reflected by the slope of the time-course curve of [AO]) was evaluated using the detected aldicarb oxime concentration [AO] at a given time point (5 h for the data in Figure 3D) divided by the time.

The obtained Coomassie Blue-stained SDS electrophoresis gel of the purified CocH3-Fc(M3) protein shown in Figure 3B confirmed that the native structure of the purified CocH3-Fc(M3) protein was a dimer. Based on the time-course data depicted in Figure 3C, further enzymatic reactions for Michaelis–Menten kinetic analysis were carried out with 0.1 μM CocH3-Fc(M3) against aldicarb at various initial aldicarb concentrations, and all the reactions were stopped at 5 h for sampling and LC-MS/MS analysis. Shown in Figure 3D are the obtained *in vitro* activity data for Michaelis–Menten kinetics, demonstrating that k_cat_ = 0.060 min^−1^ and K_M_ = 2.5 μM. Hence, k_cat_/K_M_ (catalytic efficiency) = 2.4 × 10^4^ min^−1^ M^−1^, as summarized in Table 2.

**Table 2:** Kinetic parameters (Mean ± SD) obtained from Michaelis–Menten analysis for CocH3-Fc(M3)-catalyzed hydrolysis of aldicarb at 37°C

Enzyme	*k*_cat_ (min^−1^)	*K*_M_ (μM)	*k*_cat_/*K*_M_ (min^−1^ M^−1^)
CocH3-Fc(M3)	0.060 ± 0.013	2.5 ± 0.4	2.4 ± 0.5 × 10^4^

### Reaction rate for converting aldicarb to AO at a higher enzyme concentration

**Figure 4:**
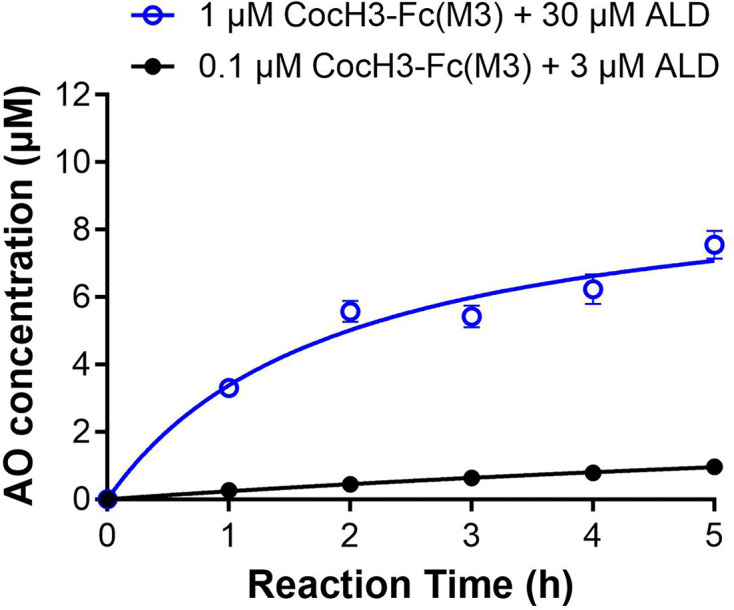
Time course of AO concentration in presence of CocH3-Fc(M3) Rate of enzyme-catalyzed detoxification of ALD is enzyme concentration dependent, as evidenced by measured [AO] versus time curves from 30 μM aldicarb hydrolyzed by 1 μM CocH3-Fc(M3) (blue) in comparison with 3 μM aldicarb hydrolyzed by 0.1 μM CocH3-Fc(M3) (black).

The *in vitro* activity data shown in Figure 3 were all based on the use of a low concentration (0.1 μM) of CocH3-Fc(M3) for convenient detection of the hydrolysis product, as the low enzyme concentration and a longer reaction time allowed us to detect and quantify the aldicarb and AO concentrations more accurately during the reaction process. However, we also wished to confirm that increasing the enzyme concentration (while keeping [enzyme] << [ALD]_0_) would increase the aldicarb hydrolysis reaction rate in order to further validate the purported catalytic activity of CocH3-Fc(M3). For this purpose, we performed an additional *in vitro* activity experiment with 1 μM CocH3-Fc(M3) against 30 μM aldicarb. The observed concentrations of AO at various time points for 1 μM CocH3-Fc(M3) + 30 μM aldicarb are shown in Figure 4 in comparison with the corresponding AO concentrations for 0.1 μM CocH3-Fc(M3) + 3 μM aldicarb. Based on the *in vitro* activity data shown in Figure 4, increasing the concentration of CocH3-Fc(M3) proportionally increased the AO production, which is consistent with the expected catalytic activity of CocH3-Fc(M3) for aldicarb hydrolysis.

## Discussion

Our previous study revealed that, as a promising cocaine hydrolase for treatment of cocaine use disorder, CocH3-Fc(M3) can be inhibited rapidly by aldicarb and that the aldicarb-inhibited enzyme CocH3-Fc(M3) can be reactivated spontaneously [23], implying that CocH3-Fc(M3) may hydrolyze aldicarb through a catalytic mechanism similar to CocH3-Fc(M3)-catalyzed hydrolysis of cocaine. As shown in Figure 3A, the binding pose of aldicarb in the active site is suitable for catalytic hydrolysis involving the known catalytic triad (formed by S198, H438, and E325) and oxyanion hole (G116, G117, and S199) of the enzyme. Particularly, within the catalytic triad, the hydroxyl oxygen of the S198 side chain is expected to gradually approach the carbonyl carbon of aldicarb to initiate the catalytic hydrolysis reaction. However, the previously published *in vitro* activity study was limited to monitoring and fitting the time course curve of the active enzyme concentration, without a method to directly detect and measure the concentrations of the hydrolysis product (AO shown in Figure 1) and the substrate (aldicarb). In the present study, by establishing an LC-MS/MS method to detect and quantify aldicarb and AO simultaneously, we were able to directly observe the CocH3-Fc(M3)-catalyzed aldicarb hydrolysis for the first time, confirming that CocH3-Fc(M3) indeed has the desirable catalytic activity for aldicarb hydrolysis and may be considered as the first aldicarb hydrolase identified so far.

Further, utilizing the developed LC-MS/MS method, we were able to carry out Michaelis–Menten kinetic analysis on the CocH3-Fc(M3)-catalyzed aldicarb hydrolysis for the first time to determine the catalytic parameters (*k*_cat_ *=* 0.060 min^−1^, *K*_M_ *=* 2.5 μM, and *k*_cat_/*K*_M_ *=* 2.4 × 10^4^ min^−1^ M^−1^). These catalytic parameters were determined at 37°C (human body temperature), which is more relevant for future *in vivo* experiments as well as translational research considerations. Notably, our previous computational simulation and fitting of the time course of the active enzyme concentration at 25°C estimated that *k*_cat_ = ∼0.995 × 10^−2^ min^−1^, *K*_M_ = ∼0.45 μM, and *k*_cat_/*K*_M_ = ∼2.21 × 10^4^ min^−1^ M^−1^ at 25°C [23]. So, the directly determined *k*_cat_ of 0.060 min^−1^ at 37°C is ∼6-fold higher than the previously estimated *k*_cat_ at 25°C, and the directly determined *K*_M_ of 2.5 μM at 37°C is ∼6-fold higher than the previously estimated *K*_M_ at 25°C. Interestingly, the directly determined catalytic efficiency (*k*_cat_/*K*_M_ *=* 2.4 × 10^4^ min^−1^ M^−1^) at 37°C is nearly identical to the previously estimated catalytic efficiency (*k*_cat_/*K*_M_ = ∼2.21 × 10^4^ min^−1^ M^−1^). As is well known, the reaction temperature change could affect both *k*_cat_ and *K*_M_ and, hence, it is possible that increasing the enzymatic reaction temperature from 25°C to 37°C has increased both the *k*_cat_ and *K*_M_ without a large change in the overall catalytic efficiency. For this reason, the previously used computational approach (involving fitting the active enzyme concentration time course) may be reasonable for a quick estimation/prediction of catalytic parameters for aldicarb hydrolysis, particularly during screening of enzymes for aldicarb hydrolysis activity. However, any enzymes identified as potential “leads” from screening efforts must be investigated further with studies involving direct detection of the hydrolysis product (as presented in this work) in order to confirm their actual catalytic activity and precisely evaluate the associated kinetic constants.

With the catalytic parameters at 37°C now determined, we can better understand the results from previous *in vivo* rescue experiments using 100, 50, and 25 mg/kg CocH3-Fc(M3) to rescue mice injected with a lethal dose of 0.75 mg/kg aldicarb (LD_100_) [23]. In the case of aldicarb poisoning in rodents, the aldicarb concentration may reach as high as ∼30 μM (example: ∼28.8 μM or 5.48 mg/l [35]) when the animals succumb to aldicarb poisoning. Similarly, for humans, the blood concentration of aldicarb reached ∼32.6 μM (6.2 mg/l) in a case report of fatal aldicarb poisoning [36] and ∼16.9 μM (3.22 mg/l) in another case report of nonfatal aldicarb poisoning [37]. So, for rescue, one might have to rapidly decrease the blood aldicarb concentration by approximately one-half to be effective. It is well-known that when the substrate concentration [S] >> *K*_M_, the catalytic reaction rate will approach V_max_ = *k*_cat_[E]. The initial plasma concentrations of CocH3-Fc(M3) in rodents were estimated to be ∼22.4, ∼11.2, and ∼5.61 μM associated with the enzyme doses 100, 50, and 25 mg/kg, respectively, for the intravenous (IV) administration of the enzyme [23]. So, using the now-determined *k*_cat_ at 37°C, V_max_ = 1.3, 0.65, and 0.33 μM min^−1^ for the doses 100, 50, and 25 mg/kg, respectively. V_max_ = 1.3 μM min^−1^ means that the enzyme associated with the dose of 100 mg/kg CocH3-Fc(M3) may convert 1.3 μM aldicarb to AO per minute or convert up to ∼16.3 μM aldicarb (one-half of ∼32.6 μM) to AO in ∼12.5 min. In other words, the high dose of 100 mg/kg CocH3-Fc(M3) would need ∼12.5 min to decrease the reported lethal blood aldicarb concentration by one-half, and would need ∼25 and ∼50 min to decrease the high blood aldicarb concentration by one-half with the enzyme doses of 50 and 25 mg/kg, respectively. 50 min might be too long for the rescue, which explains why 25 mg/kg CocH3-Fc(M3) only provided partial rescue efficacy while both the 100 and 50 mg/kg CocH3-Fc(M3) provided the desired full rescue efficacy.

This study has not only confirmed that CocH3-Fc(M3) is indeed an aldicarb hydrolase suitable for aldicarb poisoning treatment and determined its catalytic parameters at 37°C for the first time, but also provided a generally useful LC-MS/MS method to detect and quantify aldicarb and its hydrolysis product AO for further studies on aldicarb hydrolysis. Due to intensive interests in food and environmental safety as well as aldicarb-related toxicology studies, particularly in the detection of aldicarb [9–21], the LC-MS/MS method developed in this study may also be used as a valuable tool to quantitatively and accurately detect aldicarb and its reaction products in future food and environmental safety control efforts and aldicarb-related toxicology studies. Additionally, the obtained kinetic data and LC-MS/MS method will also be valuable for further *in vivo* studies and translational research using CocH3-Fc(M3), as well as for designing more potent enzymes to hydrolyze aldicarb in the future.

## Materials and methods

### Materials

CocH3-Fc(M3) protein was expressed and purified as described in our previous report [25]. Briefly, a highly efficient stable Chinese Hamster Ovary (CHO)-S cell line, generated by a lentivirus-based method [28,38] to express CocH3-Fc(M3) [25], was used for the protein expression with a base media containing Gibco FreeStyle™ CHO expression media supplemented with 100 U/ml penicillin, 100 μg/ml streptomycin, and 8 mM L-glutamine. Once the seed vial was thawed, cells were pelleted and resuspended in complete media, then grown in adherent culture for 72 h in the base media + 10% fetal bovine serum (FBS) at temperature of 37°C and 5% CO_2_. After the confluence was achieved, adhered cells were suspended by adding trypsin, pelleted, resuspended in base media, and introduced into suspension culture containing base media and 3% FBS, using shake flasks and orbital shaker at a speed of 125 RPM, temperature of 37°C, and 5% CO_2_ for further cell expansion to obtain suffiicent amount of cells so as to inoculate enzyme expression cultures (base media and orbital shaker at a speed of 125 RPM, 37°C, and 5% CO_2_) with an initial density of 5 × 10^5^ viable cells/ml. Two temperature shifts were applied during the protein production: Day 2 (shifting the temperature to 32°C) and Day 4 (shifting the temperature to 30°C). The culture media was harvested when VCD (viable cell density) ≤90%, and the secreted enzyme was purified from clarified media using the well-known Protein A affinity chromatography as described in detail in our previous report [25]. The purified protein was dialyzed in storage buffer and stored at −80°C before use. The use of Protein A affinity chromatography protocol for our Fc-fusion proteins has consistently resulted in a high purity of >90% according to our previous studies [28,39,40]. The enzyme concentration was calibrated by both the well-known Bradford assay and our sensitive radiometric [^3^H](-)-cocaine hydrolysis assay (with the previously known catalytic parameters against (-)-cocaine: k_cat_ = 5700 min^−1^ and K_M_ = 3.1 at 25°C for CocH3-Fc(M3) against (-)-cocaine) [28]. Both experimental protocols resulted in comparable concentration results with a difference ≤20%. As the radiometric cocaine hydrolysis assay can be used to detect the active enzyme concentration in a complex mixture and is consistent with our previous studies, in this study the CocH3-Fc(M3) concentration [E] was evaluated by this method; specifically, by determination of the cocaine hydrolysis rate under saturating conditions and applying [E] = V_max_/k_cat_.

Aldicarb was ordered from Sigma–Aldrich (St. Louis, MO). AO and the isotopically labeled IS, including aldicarb-d3 and aldicarb sulfone-d3, were purchased from Dr Ehrenstorfer Reference Materials (Augsburg, Germany; https://www.lgcstandards.com/GB/en/). Paraoxon, HPLC-grade methanol, bovine serum albumin (BSA) powder, acetonitrile, and all materials for electrophoresis experiment were purchased from Thermo Fisher Scientific (Waltham, MA). Formic acid was ordered from Sigma–Aldrich (St. Louis, MO). The other general supplies were purchased from Thermo Fisher Scientific (Waltham, MA), Sigma–Aldrich (St. Louis, MO), or VWR International (Radnor, PA).

### Electrophoresis

Sodium dodecyl sulfate–polyacrylamide gel electrophoresis (SDS–PAGE) was conducted in precast Novex 4–12% Tris-Glycine Plus Mini Gels (8.0 cm × 8.0 cm × 1.0 mm). Enzyme CocH3-Fc(M3) was pre-diluted to 0.1 mg/ml in 0.1 M phosphate buffer (PB). A nominal enzyme amount of 1 μg was loaded per enzyme well. Enzyme lane loading materials were prepared by combining pre-diluted enzyme with concentrated sample buffer and MilliQ water with final loading volume of 40 μl. For non-reducing conditions, pre-diluted enzyme was combined with Thermo Fisher Scientific Non-Reducing Lane Marker Sample Buffer. For reducing conditions, pre-diluted enzyme was combined with Thermo Fisher Scientific Reducing Lane Marker Sample Buffer and 5% 2-mercaptoethanol, then incubated in near-boiling water for 5 min. The following running buffer was used: 25 mM Tris, 192 mM glycine, and 0.1% SDS in MilliQ water. Electrophoresis was run in an XCell SureLock Mini-Cell chamber under a constant voltage of 100 V (12.5 V/cm for 8 cm length Novex mini gels) for 2 h.

After electrophoresis, the gel was removed from its cassette and subjected to Coomassie staining by submersion in staining solution (0.3% Coomassie Brilliant Blue R-250 dye, 10% acetic acid, 40% methanol, aqueous) for 1 h with inversion of gel halfway through staining, followed by submersion in destaining solution (10% acetic acid, 40% methanol, aqueous) for 30 min, submersion in fresh destaining solution for additional 30 min, then submersion in fresh destaining solution overnight. All staining/de-staining steps were conducted with gentle agitation via plate shaker. The gel was imaged in shallow de-staining solution using a Bio-Rad Gel Doc XR+ Imaging System chamber and Bio-Rad Image Lab software (version 6.0.1).

The high purity of the purified CocH3-Fc(M3) in this study was evidenced by the analytical results of SDS–PAGE electrophoresis of the enzyme shown in Figure 3B.

### Instruments and conditions for the LC-MS/MS protocol

A Shimadzu HPLC system (Shimadzu, Kyoto, Japan) cascaded with a mass spectrometer AB SCIEX Triple TOFTM5600 (AB SCIEX, Redwood City, CA, USA) was used in this study. Analyst^®^ TF 1.7 software (AB SCIEX, Redwood City, CA, USA) was used for instrument control and data acquisition [41,42].

Preparation of stock, calibration standards, and quality control samples. For LC-MS/MS method development and analysis, individual stock solutions were prepared at 10 mg/ml for each compound and each IS in methanol. Then, a mixed analyte stock solution—composed of aldicarb and AO—was prepared by mixing and diluting stock solutions to 1 mg/ml in methanol, and a mixed IS stock solution was prepared by mixing and diluting aldicarb-d3 and aldicarb sulfone-d3 stock solutions to 50 μg/ml in methanol. This mixed IS solution was further diluted to 2500 ng/ml in Milli-Q water, as well as to 1000 ng/ml in Milli-Q water containing 2 mM paraoxon. A combined stock solution was then prepared at 500 μg/ml for each analyte (using the 1 mg/ml combined analytes solution) and 1000 ng/ml for each IS (using the 2500 ng/ml combined IS solution) with 2 mM paraoxon in Milli-Q water. Finally, working standard solutions were prepared by diluting the combined stock solution to various concentrations (10000, 5000, 2000, 1000, 500, 250, 100, 20, and 10 ng/ml) using the 1000 ng/ml IS + 2 mM paraoxon solution (fixed concentration for IS). All stock and working standard solutions were stored at −20°C until use. Calibration standard samples were prepared by combining 50 μl of different concentrations of working standard solutions with 50 μl of *in vitro* assay vehicle buffer (0.1 M PB with 1 mg/ml BSA, pH = 7.4), in order to match the final concentrations of all non-analyte components from the in vitro kinetics assays (see “*In vitro* activity assays” section below). Finally, to remove protein, these calibration standard samples were processed with Amicon 0.5 ml 30 kDa MWCO centrifuge filters (13000 RCF × 20 min at 20°C) with flow-through retained. All calibration curves were established using standards in the *in vitro* buffer. Each calibration curve was prepared in three replicates. A volume of 20 μl for each sample was injected for LC-MS/MS analysis.

Optimization of liquid chromatographic and mass spectrometric conditions. Chromatographic separation was carried out on a reversed-phase C18 column (Atlantis T3, 100 Å, 3 μm, 2.1 mm × 150 mm I.D., Waters, Milford, MA, U.S.A.) with a security guard cartridge kit (Atlantis T3 VanGuard cartridge plus holder, Waters) using a gradient mobile phase composed of 0.1% formic acid in Milli-Q water (phase A) and acetonitrile (phase B). The following gradient was set for mobile phase: 5% B at 0 min, hold 5% B for 3 min, then B increased to 20% at 5 min, 50% at 13 min, and 95% B at 17 min, hold 95% B for 4.5 min, 5% B at 23.5 min, hold 5% for 0.5 minute. The total sample run time was 24 min/sample, and re-equilibrated at 5% B for 4 minutes before the next sample was injected. The column was kept at room temperature (∼21°C). The samples and autosampler temperature were maintained at 10°C during sample acquisition, and the autosampler injection needle was washed with 300 μl of 50% mobile phase A in methanol after each sample injection to reduce carryover. The flow rate was set at 0.2 ml/min and the injection volume for each sample was 20 μl. The mass spectrometer was run in high sensitivity and positive ion mode with nitrogen as the source gas, and compound-specific ionization parameters—including ion transition, declustering potential (DP), collision energy (CE), collision energy spread (CES), ion release delay (IRD), and ion release width (IRW)—were optimized individually by running syringe injection at 10 μg/ml in methanol and summarized in Table 3. By determining the concentrations of these compounds in the samples collected at various time points after injection of the compound(s), this method allowed us to uncover the biochemical reactions that occurred in the *in vitro* assays.

**Table 3:** Optimized compound-specific source parameters for the electrospray ionization including ion transition, DP, CE, CES, IRD, and IRW.

Compounds	Ion transition	DP(V)	CE(V)	CES(V)	IRD	IRW	Retention time (min)
ALD	208.1→116.1	50	10	3	67	20	16.00
AO	134.1→86.1	33	10	3	67	20	15.22
ASF-d3	226.1→148.1	33	13	3	67	20	11.26

### *In vitro* activity assays

For detection and quantification of the hydrolysis of ALD (resulting in formation of the hydrolysis product AO), enzyme CocH3-Fc(M3) was incubated with ALD in 0.1 M PB + 1 mg/ml BSA (pH 7.4) at 37°C, with the enzyme concentration [E] = 0, 0.1 μM (i.e. 100 nM), or 1 μM and [aldicarb] = 1, 3, 5, 7, 10, 25, 30, or 50 μM. At multiple timepoints of 1, 2, 3, 4, and/or 5 h within 5 h of reaction of ALD with CocH3-Fc(M3), the aldicarb-enzyme reaction mixture was sampled, and the samples were immediately quenched with an equivalent volume of an aqueous solution containing 2 mM paraoxon-ethyl (for enzyme inactivation) and 1000 ng/ml IS. To remove reaction mixture proteins from the sample (enzyme and BSA), quenched samples were processed with Amicon 0.5 ml 30 kDa MWCO centrifuge filters (13000 RCF × 20 min at 20°C), and the flow-through was collected and kept frozen at −20°C until ready for measurement. The sample analyte concentrations were then determined after LC-MS/MS measurement by integrating the peak areas of analytes and the IS.

### Data analysis

Concentrations of ALD and AO were determined using the developed LC-MS/MS method. All the concentrations were analyzed using MultiQuant™ 3.0 software (AB SCIEX, Redwood City, CA) with the unit of nanogram/milliliter in accordance with the prepared standard curves.

The *in vitro* activity assays for Michaelis–Menten analysis were performed in triplicate, and the obtained reaction rates were analyzed according to the Michaelis–Menten kinetics that allowed us to determine the kinetic parameters (V_max_ and *K*_M_ values). The other *in vitro* activity assays were carried out in duplicate. Based on the determined V_max_ and the enzyme concentration [E] used, the catalytic rate constant *k*_cat_ can be evaluated: *k*_cat_ = V_max_/[E]. The Michaelis–Menten curve and time-dependent concentration curves of analytes were plotted using Prism 10 software (GraphPad Software, San Diego, CA), which was also used for all other data analysis after calculation of [ALD] or [AO] using the MultiQuant™ software. Data in all the figures were presented as the mean and the standard error of the mean (SEM).

## Data Availability

Data will be made available on reasonable request.

## Open Access

Open access for this article was enabled by the participation of the University of Kentucky in an all-inclusive Read & Publish agreement with Portland Press and the Biochemical Society.

## Abbreviations

AChE: acetylcholinesterase
ALD: aldicarb
AO: aldicarb oxime
ASF: aldicarb sulfone
BChE: butyrylcholinesterase
BSA: bovine serum albumin
CHO: Chinese Hamster Ovary
CE: collision energy
CES: collision energy spread
DP: declustering potential
FBS: fetal bovine serum
ICU: intensive care unit
IS: internal standard
IV: intravenous
IRD: ion release delay
IRW: ion release width
LLOQ: lower limit of quantification
LOD: limit of detection
MMBC: Molecular Modeling and Biopharmaceutical Center
OP: organophosphate
PB: phosphate buffer
SDS-PAGE: sodium dodecyl sulfate polyacrylamide gel electrophoresis
ULOQ: upper limit of quantification
VCD: viable cell density
